# Identification and Evaluation of Reference Genes for Normalization of Gene Expression in Developmental Stages, Sexes, and Tissues of *Diaphania caesalis* (Lepidoptera, Pyralidae)

**DOI:** 10.1093/jisesa/iez130

**Published:** 2020-01-11

**Authors:** Zheng Wang, Qianqian Meng, Xi Zhu, Shiwei Sun, Aiqin Liu, Shengfeng Gao, Yafeng Gou

**Affiliations:** 1 Hainan Provincial Key Laboratory of Genetic Improvement and Quality Regulation for Tropical Spice and Beverage Crops, Spice and Beverage Research Institute, Chinese Academy of Tropical Agricultural Sciences, Wanning, China; 2 Institute of Tropical Agriculture and Forestry, Hainan University, Haikou, China

**Keywords:** *Diaphania caesalis* (Walker), reference gene, developmental stages, tissue, sexes

## Abstract

*Diaphania caesalis* (Walker) is an important boring insect mainly distributed in subtropical and tropical areas and attacked tropical woody grain crops, such as starchy plants of Artocarpus. Quantitative real-time polymerase chain reaction (qRT-PCR) is a powerful approach for investigating target genes expression profiles at the transcriptional level. However, the identification and selection of internal reference genes, which is often overlooked, is the most vital step before the analysis of target gene expression by qRT-PCR. So far, the reliable internal reference genes under a certain condition of *D. caesalis* have not been investigated. Therefore, this study evaluated the expression stability of eight candidate reference genes including ACT, β-TUB, GAPDH, G6PDH, RPS3a, RPL13a, EF1α, and EIF4A in different developmental stages, tissues and sexes using geNorm, NormFinder and BestKeeper algorithms. To verify the stability of the recommended internal reference genes, the expression levels of *Dcae*OBP5 were analyzed under different treatment conditions. The results indicated that ACT, RPL13a, β-TUB, RPS3a, and EF1α were identified as the most stable reference genes for further studies on target gene expression involving different developmental stages of *D. caesalis*. And ACT and EIF4A were recommended as stable reference genes for different tissues. Furthermore, ACT, EF1α, and RPS3a were ranked as the best reference genes in different sexes based on three algorithms. Our research represents the critical first step to normalize qRT-PCR data and ensure the accuracy of expression of target genes involved in phylogenetic and physiological mechanism at the transcriptional level in *D. caesalia*.

Quantitative real-time polymerase chain reaction (qRT-PCR) is widely used for analyzing expression of specific genes because of the advantages of high sensitivity, accuracy, specificity, and rapidity (Shu et al. 2018, Wang et al. 2018). Moreover, this technique has also promoted research process in entomology, particularly for analyzing the time-specific expression of target genes and detecting low-abundance transcripts (Guénin et al. 2009). However, the accuracy and reliability of qRT-PCR results are severely affected by many variations, such as true biological signals, amplification efficiency of primers, stability of reference genes, the quantity and quality of templates used, the yield of the extraction process, and differences in the enzymatic reactions (Jiang et al. 2015, Wang et al. 2015a, Hu et al. 2018, Zhang et al. 2018). Among them, normalization has been considered as one of the greatest influencing factor. In order to minimize the effect of these variations, the application of reference genes with stable expression, which is often overlooked, is an essential prerequisite for the precise normalization of qRT-PCR results (Shekh et al. 2017). The guidelines should ensure the selection of reference genes to be transcribed are constitutively the same level in all samples, regardless of what developmental stages, tissues or other experimental conditions are used, furthermore, multiple reference genes were strongly recommended to be employed for normalization (Appukuttan et al. 2018, Shakeel et al. 2018).

In many studies of gene functional analysis, housekeeping genes including actin (ACT), glyceraldehyde-3-phosphate dehydrogenase (GAPDH), 18S ribosomal RNA (18S rRNA), elongation factor 1 alpha (EF1α), and β-tubulin (TUB) have been utilized heavily as the internal control in many different species without proper validation (Suzuki et al. 2000, Xu et al. 2017, Zhang et al. 2017). The premise of these practices have tacitly approved that these reference genes were expressed consistently throughout different test conditions. Actually, no one gene has been found to be stably expressed across different treatments within a single species, not to mention in different species (Pan et al. 2015, Prunier et al. 2016, Yang et al. 2016, Najat et al. 2018, Li et al. 2018). For example, in *Thitarodes armoricanus* (Lepidoptera: Hepialidae), GAPDH was verified to be the best reference gene suited for normalization in different developmental stages and larvae body parts, while it was not suitable under lower-temperature challenge, fungal infections, and dietary treatments (Liu et al. 2016). Moreover, the classical reference gene ACT was verified as the most suitable reference gene at different developmental stages in *Aedes albopictus* (Diptera, Culicidae). But it was not suitable for different developmental stages of *Helopeltis theivora* (Waterhouse) (Hemiptera, Miridae) (Dzaki et al. 2017, Wang et al. 2019). Recent studies found that some novel reference genes, such as ribosomal protein genes RP49, RPS15, RPL13, and RPS9, were verified as a set of potential internal control genes (Teng et al. 2012, Barros Rodrigues et al. 2014, Zhang et al. 2015). Besides, some research pointed out the combination use of two or more reference genes could be demanded when a single reference gene cannot meet the experimental requirements (Tong et al. 2009, Chen et al. 2011, Shi et al. 2016). Therefore, to avoid misinterpretation of gene expression data, it is essential to check the expression stability of reference genes before normalizing target gene expression (Lu et al. 2015, Shakeel et al. 2015).

Jackfuit borer, *Diaphania caesalis* (Walker), is an important boring insect mainly distributed in subtropical and tropical areas and attack tropical characteristic woody grain crop, such as *Artocarpus heteroyllus* (Lam.), *Artocarpus champeden* (Spreng.), and *Artocarpus altilis* (Fosberg.). The larva does harm directly inside the tender shoots, floral buds, and fruits, which tend to cause fruit rots rendering the fruit commercially worthless. However, due to lack of a stably expressed reference gene for the accurate normalization of qRT-PCR data, the studies on the molecular biology in *D. caesalis*, including the functional study of target genes and physiological mechanisms of its adaptability, are not clear, which has been regarded as a major hurdle for deeper studies of this species.

To obtain suitable reference genes under different development stages, tissues, and sexes of *D.caesalis*, eight candidate reference genes including ACT, β-TUB, GAPDH, Glucose-6-phosphate 1-dehydrogenase (G6PDH), 40S ribosomal protein S3 a (RPS3a), 60S ribosomal protein L13 a (RPL13a), EF1α and eukaryotic initiation factor 4A (EIF4A) were identified and the expression stabilities were evaluated by several statistical models such as softwares geNorm, NormFinder, and BestKeeper (Liu et al. 2016). To further validate our results, the expression profiles of the gene encoding odorant binding protein-5 (*Dcae*OBP5) were analyzed under different conditions. The reference genes identified in this study will facilitate future studies on gene expression in this important pest species.

## Materials and Methods

### Insect Rearing

Larvae of *D. caesalis* were collected from jackfruit in the field of Spice and Beverage Research Institute, Chinese Academy of Tropical Agricultural Sciences, reared in mesh cages and fed on jackfruit leaves in the laboratory. The adults were provisioned 10% sucrose solution after eclosion. A laboratory colony was established and maintained at 26 ± 1°C, 75 ± 5% relative humidity and 14:10 (L:D) h cycle.

### Sample Collection

#### Development Stages

Five samples including larva (three individuals), female pupa (three individuals), male pupa (three individuals), female adults (three individuals), and male adults (three individuals), were separately collected for stability evaluation of reference genes. No obvious mortality was observed during the sample collection.

#### Tissues

Six tissue samples (head, antenna, thorax, abdomen, legs and wings) were dissected from healthy male and female adults using sterilized scalpel and tweezers. Each tissue was collected from 20 adults (half males and half females) 2 or 3 d following emergence.

#### Sexes

Eight samples were taken from each sex, including pupa, adults, head, antenna, thorax, abdomen, legs, and wings, and were prepared for reference gene selection. In detail, each pupa or adult samples contained material from three active individuals, respectively, and tissue samples in different sexes were collected from 20 adults 2 or 3 d after emergence.

All the samples were flash-frozen in liquid nitrogen and stored at −80°C until used. Each sample was repeated in triplicate.

### Total RNA Extraction and cDNA Synthesis

Total RNA of each sample was extracted by TRIzol Reagent (Invitrogen, United States) following the manufacturer’s protocol. Each sample was homogenized with 1 ml TRIzol reagent. A moderate amount of RNase-free water was added to dissolve the precipitate. The concentration and purity of total RNA were measured by fluorescence microplate reader (BioTek, United States). The first-strand cDNA was synthesized by FastKing RT kit (with gDNase) (TIANGEN, China) in accordance with the manufacturer’s instructions. The protocol was as follows: the first step was to remove the gDNA with 10 µl reaction system contained 1 µg of total RNA, 2 µl of 5×gDNase and additional RNase-free water. The mixture was incubated at 42°C for 3 min and chilled in the ice immediately. The second step was prepared another 10 µl reaction solution with 2 µl 10×King RT Buffer, 1 µl FastKing RT Enzyme Mix, 2 µl FQ-RT Primer Mix, and 5 µl RNase-free water, then added it to the solution above. Make sure the total volume of the admixture was up to 20 µl. The reaction procedure was performed at 42°C for 15 min, 95°C for 3 min. The products were stored at −20°C before use.

### Candidate Reference Genes Selection and Primer Design

Eight commonly used reference genes, separately encoding ACT, β-TUB, GAPDH, G6PDH, RPS3a, RPL13a, EF1α, and EIF4A were selected from *D. caesalis* transcriptome sequencing data as candidate genes. The primers were designed by NCBI based on the sequence of each gene. The details of the primers are shown in Table 1.

**Table 1.** Primer amplification characteristics of eight candidate genes for qRT-PCR in *D. caesalis*

Gene name (abbreviation)	Accession number	Primer sequence	Amplification size (bp)	Amplification efficiency (%)	Correlation coefficient (*R*^*2*^)
ACT	MN062588	5′ ACAATGAACTCCGTGTCGCC 3′ 5′ GTACATGGCGGGTGTGTTGA 3′	128	102.9	0.998
β-TUB	MN062589	5′ GGGAACGCTCCTCATCTCAA 3′ 5′ AGAGTGGCGTTGTAGGGTTC 3′	120	95.1	0.999
GAPDH	MN062590	5′ TCGTTGATCTCACTGTCCGC 3′ 5′ TTCGGTGTATCCAAGGACGC 3′	110	96.5	0.999
G6PDH	MN062591	5′ CCGATTTCCAACATCCGCAC 3′ 5′ CACTTTTCTGGCGCACATCC 3′	177	98	0.999
RPS3a	MN062592	5′ TGAACATGGCGGTCGGTAAA 3′ 5′ ATTGAACATGGACGGTGCCT 3′	125	99.2	0.999
RPL13a	MN062593	5′ CCGTGGACCTTTCCACTTCA 3′ 5′ ACTTCCAGCCGACTTCATGG 3′	245	107.5	0.998
EF1α	MN062594	5′ ATGGTTCAAGGGATGGCTCG 3′ 5′ GTTTCGACTCTGCCTACGGG 3′	180	98	0.998
EIF4A	MN062595	5′ ATGGGCCAAAGGATCAAGGG 3′ 5′ TGGGCTATAACATCGCGTCC 3′	211	106.4	0.998

### RT-PCR and qRT-PCR Analysis

RT-PCR amplifications of these eight pairs of primers were performed with *Premix Taq* (*TaKaRa Taq* Version 2.0 plus dye) by the following program: denaturing at 94°C for 3 min, followed by 35 cycles at 94°C for 30 s, 60°C for 30 s and 72°C for 1 min, with a final extension at 72°C for 10 min. The amplification products were detected by 1.5% agarose gel electrophoresis and extracted by E.Z.N.A. Gel Extraction Kit (Omega, United States). The fragments were ligated to pMD-19T and transformed into *Escherichia coli* DH5α (TaKaRa, China). Plasmids were extracted by E.Z.N.A. Plasmid Miniprep Kit II (Omega, United States) and used as the templates for standard curve generation of the candidate genes.

qRT-PCR reactions were fulfilled by iTaq Universal SYBR Green Supermix (Bio-Rad, China) on BioRad CFX96 Real-Time PCR detection system. Each reaction system was designed with three technical replicates. Amplification condition was performed by a denaturation step at 95°C for 3 min, followed by 40 cycles of 95°C for 10 s and 60°C for 30 s. Following the reaction, a melting curve analysis from 65°C to 95°C was applied to ensure consistency and specificity of each amplified product. A series of 10-fold dilution of plasmids were used to create the five-point standard curves by a linear regression model (Pfaffl et al. 2004). The regression equation was carried out to calculate the efficiency (E) and correlation coefficient (*R*^*2*^) of each primer pair. The efficiencies (E) of corresponding primers were estimated according to the equation: E = (10^[–1/slope]^ −1) × 100.

### Validation of Reference Genes

To evaluate the validity of selected reference genes, the transcription levels of *Dcae*OBP5 were estimated in different development stages and tissues. qRT-PCR amplification of *Dcae*OBP5 (Accession number: MN062600) was performed with primers as follows: Forward (GTGTGCATTGGAACTGAGCG) and Reverse (TTTATGTCCTCCTCCGCGAC). The relative expression levels of *Dcae*OBP5 were determined according to the Ct values based on the 2^*−*ΔΔCt^ method (Livak and Schmittgen 2001). All the treatments were performed in three biological and technical replicates, respectively. One-way ANOVA was used to compare the effects of treatments. Statistical analyses were performed using SPSS 20.0 (SPSS, Inc., United States)

### Statistical Analysis

The cycle threshold values (Ct values) from qRT-PCR were collected, and the stability of candidate reference genes were ranked by the software tools of geNorm, NormFinder, and BestKeeper. The relative quantities converted from the raw Ct values (the highest relative quantity of gene was set to 1) were used as input data for geNorm and NormFinder. geNorm calculates the expression stability value (M) and pairwise variation (V). Gene expression is considered stable when the M value is below 1.5, and the lower the M values, the more stable the expression. Besides, the pairwise variation (V_n_/V_n+1_) is used to determine the optimal number of reference genes. The threshold of V_n_/V_n+1_ is 0.15. The V_n_/V_n+1_ value below 0.15 suggested that an extra reference gene is not required for normalization (Vandesompele et al. 2002). NormFinder algorithm ranks the candidate reference genes based on the evaluation of both intra- and inter-group variation and a separate analysis of the sample subgroups in expression levels (Andersen et al. 2004). The stability of candidate reference genes evaluated by BestKeeper applet depends on the SD of the raw Ct values. The lower SD value means the gene is more stable (Pfaffl et al. 2004).

## Results

### Amplification Specificity and Efficiency of Candidate Reference Genes

**Fig. 1.**
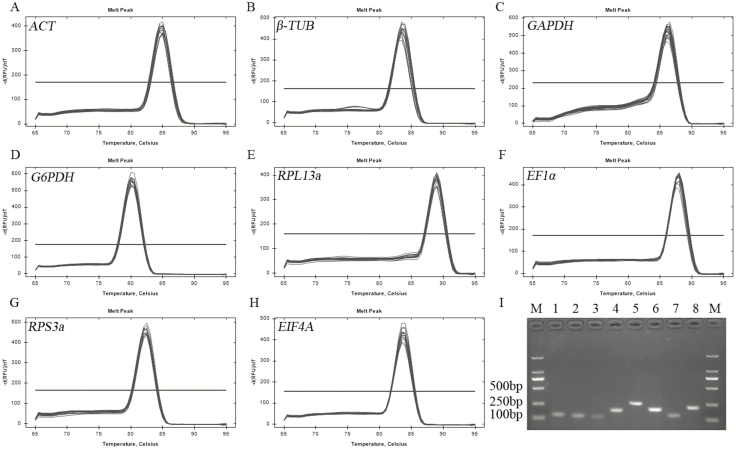
Amplification specificities of eight candidate reference gene primers in qRT-PCR and RT-PCR. (A–H) Melt curve analysis of eight candidate genes. (I) Agarose gel electrophoresis results of PCR products amplified by eight specific primer pairs. Lane M, DNA 2000 Marker, and lane 1–8, ACT, β-TUB, GAPDH, G6PDH, RPL13a, EF1α, RPS3a, EIF4A.

To evaluate the stability of the eight candidate reference genes, the amplification specificity and efficiency of eight specific primer pairs were identified first. The results showed that all the PCR products amplified by the primers were observed as a single band with expected strip size ranging from 110 to 245 bp on 1.5% agarose gel (Fig. 1I). In addition, the amplification specificities of all the primer pairs were illustrated by a single peak in melting curve analysis (Fig. 1A–H). The efficiency (E) and correlation coefficient (*R*^*2*^) of all the primers were calculated and shown in Table 1. The amplification efficiencies of primers met the standard requirement of conventional qRT-PCR, which was confirmed by primer efficiency ranging from 95.1 to 107.5% and almost all the standard curves of *R*^2^ > 0.998.

### Comparative Expression Levels of Candidate Reference Genes

**Fig. 2.**
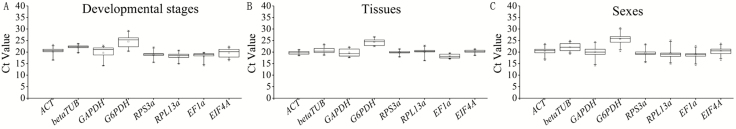
Average Ct values of eight candidate reference genes at developmental stages (A), tissues (B), and sexes (C) in *D. caesalis*.

The Ct values of eight candidate reference genes in 27 samples (including five samples comparing different developmental stages, six samples comparing different tissues and sixteen samples from different sexes) were detected by qRT-PCR. The average Ct values of these eight genes in each sample ranged from 14.63 to 28.06 cycles. Almost all the threshold fluorescence peaks of candidate reference genes were between 15 and 30 cycles. The dispersion of Ct values of each gene were varied among different treatments. To be specific, in developmental stages, the maximum and minimum dispersion of Ct values were identified for 7.43 cycles of G6PDH and 2.72 cycles of β-TUB (Fig. 2A). However, in tissues, the maximum dispersion was 5.89 cycles for RPL13a, followed by 4.21 cycles for β-TUB, 4.08 cycles for GAPDH, 3.69 cycles for G6PDH, 3.30 cycles for RPS3a, 2.32 cycles for EIF4A, 2.29 cycles for ACT, and the minimum dispersion was 2.15 cycles for EF1α (Fig. 2B). In sexes, the maximum and minimum dispersion of Ct values were 9.63 cycles for RPL13a and 4.51 cycles for β-TUB, respectively. The dispersions of Ct values of other genes were as follows: 9.16 for G6PDH, 7.81 cycles for EF1α, 7.69 cycles for RPS3a, 6.67 cycles for EIF4A, 6.34 cycles for ACT, and 6.09 cycles for GAPDH (Fig. 2C).

### Identification of Best Reference Gene in Developmental Stages

**Fig. 3.**
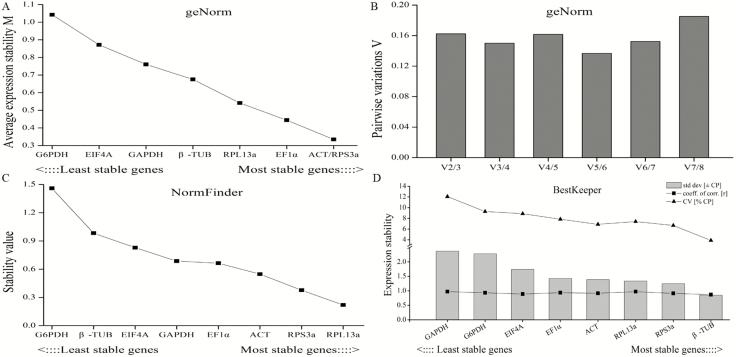
Stability analysis of candidate reference genes expression analyzed by geNorm, NormFinder and BestKeeper in developmental stages. (A) The stability M values of candidate genes by geNorm algorithm. (B) The pairwise variations (V_n_/V_n+1_) of candidate genes by geNorm algorithm. (C) The stability value of candidate genes by NormFinder algorithm. (D) The expression stability of candidate genes by BestKeeper algorithm.

In developmental stages, the M values of all the candidate reference genes calculated by geNorm were below 1.5, which means the expression levels of these genes were relatively stable. ACT and RPS3a had the lowest M value of 0.34 and were thus recommended as the best reference genes (Fig. 3A). The least stable gene was G6PDH with the highest M value of 1.04. As shown in Fig. 3B, the pairwise variations V_5_/V_6_ were less than 0.15, which suggested that five genes were necessary for more reliable normalization of target genes. Moreover, the most stable reference gene demonstrated by NormFinder analysis was RPL13a, followed by RPS3a, ACT, EF1α, GAPDH, EIF4A, β-TUB, and G6PDH (Fig. 3C). In addition, the Bestkeeper software results showed that β-TUB was identified as the best reference gene with the lowest SD value of 0.85, while the other genes were ranked as follows: RPS3a>RPL13a >ACT>EF1α>EIF4A >G6PDH >GAPDH (Fig. 3D).

### Identification of Best Reference Gene in Tissues

**Fig. 4.**
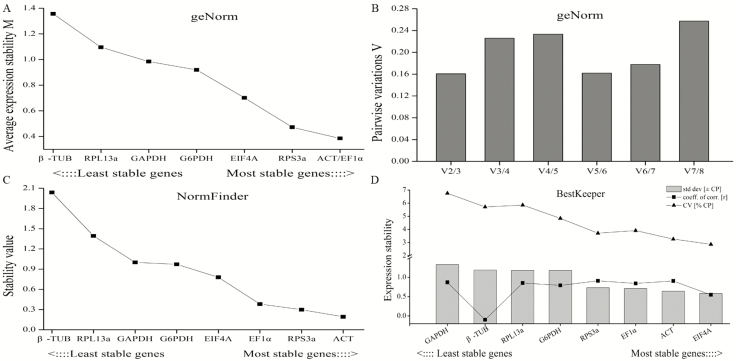
Stability analysis of candidate reference genes expression analyzed by geNorm, NormFinder and BestKeeper in tissues. (A) The stability M values of candidate genes by geNorm algorithm. (B) The pairwise variations (V_n_/V_n+1_) of candidate genes by geNorm algorithm. (C) The stability value of candidate genes by NormFinder algorithm. (D) The expression stability of candidate genes by BestKeeper algorithm.

In tissues of antenna, head, thorax, abdomen, leg, and wing, the geNorm analysis showed that the M value of all the candidate reference genes were below 1.5 (Fig. 4A). Furthermore, ACT and EF1α with the lowest M value of 0.39 were evaluated as the most stable reference genes. The least stable gene in the ranking was β-TUB. Although the pairwise variation were all above 0.15, we could confirm the number of reference genes based on the lowest V_n_/V_n+1_ score. Therefore, the V_2_/V_3_ with the lowest value of 0.16 demonstrated that two reference genes were reliable for normalization of target genes (Fig. 4B). Moreover, NormFinder software recommended ACT as the most ideal reference gene, followed by RPS3a, EF1α, EIF4A, G6PDH, GAPDH, and RPL13a based on the stability value. Again, the least stable gene identified was β-TUB (Fig. 4C). Moreover, according to BestKeeper analysis, the most and least stable reference genes in tissues were EIF4a and GAPDH, respectively. The stability ranking for the other candidate reference gene was ACT>EF1α>RPS3a> G6PDH>RPL13a >β-TUB (Fig. 4D).

### Identification of Best Reference Gene in Sexes

**Fig. 5.**
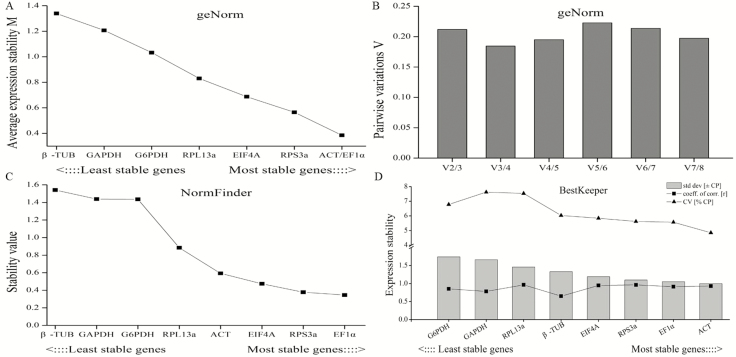
Stability analysis of candidate reference genes expression by geNorm, NormFinder and BestKeeper in sexes. (A) The stability M values of candidate genes by geNorm algorithm. (B) The pairwise variations (V_n_/V_n+1_) of candidate genes by geNorm algorithm. (C) The stability value of candidate genes by NormFinder algorithm. (D) The expression stability of candidate genes by BestKeeper algorithm.

In sexes, the M values of all the genes were below 1.5, and the most stable two genes were ACT and EF1α with the lowest M value of 0.38 based on the geNorm analysis. β-TUB with the highest M value of 1.36 was ranked as the least stable gene (Fig. 5A and B). As in the tissue sample, the pairwise variations were all above 0.15. The lowest value was V_3_/V_4_, indicating that three reference genes were recommended as internal control for normalization of target genes. Besides, the ranking of reference gene stability based on NormFinder analysis was EF1α>RPS3a>EIF4A>ACT>RPL13a>G6PDH>GAPDH>β-TUB (Fig. 5C). As shown in Fig. 5D, ACT with the lowest SD value was evaluated to be the most stable gene according to Bestkeeper analysis, followed by EF1α, RPS3a, EIF4A, β-TUB, RPL13a, GAPDH, and G6PDH.

### Comprehensive Rankings of the Three Algorithms

The comprehensive ranking of these eight reference genes following analysis by all three algorithms are shown in Table 2. The final number of suitable reference genes should integrate the results of different algorithms. Therefore, according to the pairwise variations V_n_/V_n+1_, and the criteria that the genes must be ranked in the top three genes by at least one of the algorithms, we recommended five most suitable reference genes for developmental stages: ACT, RPL13a, β-TUB, RPS3a, and EF1α. For tissues, we recommended ACT and EIF4a as the two most suitable reference genes, selected from being the top one gene from each algorithm as the best internal control. For sexes, the three suitable genes were ACT, EF1α, and RPS3a, selected from being the top two genes from each algorithm.

**Table 2.** Expression stability rankings of the eight candidate reference genes by three algorithms of geNorm, NormFinder, and BestKeeper

Experimental conditions	Methods	Stability ranking							
		1	2	3	4	5	6	7	8
Developmental stages	geNorm	ACT	RPS3a	EF1α	RPL13a	β-TUB	GAPDH	EIF4A	G6PDH
	NormFinder	RPL13a	RPS3a	ACT	EF1α	GAPDH	EIF4A	β-TUB	G6PDH
	Bestkeeper	β-TUB	RPS3a	RPL13a	ACT	EF1α	EIF4A	G6PDH	GAPDH
Tissues	geNorm	ACT	EF1α	RPS3a	EIF4A	G6PDH	GAPDH	RPL13a	β-TUB
	NormFinder	ACT	RPS3a	EF1α	EIF4A	G6PDH	GAPDH	RPL13a	β-TUB
	Bestkeeper	EIF4A	ACT	EF1α	RPS3a	G6PDH	RPL13a	β-TUB	GAPDH
Sexes	geNorm	ACT	EF1α	RPS3a	EIF4A	RPL13a	G6PDH	GAPDH	β-TUB
	NormFinder	EF1α	RPS3a	EIF4A	ACT	RPL13a	G6PDH	GAPDH	β-TUB
	Bestkeeper	ACT	EF1α	RPS3a	EIF4A	β-TUB	RPL13a	GAPDH	G6PDH

### Validation of the Selected Reference Genes

**Fig. 6.**
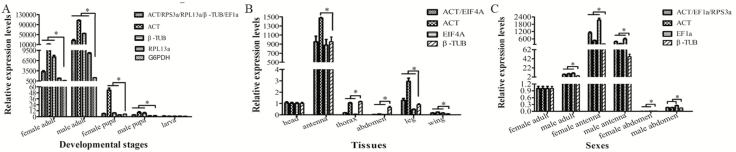
Verification of the stability of reference gene expression. Expression levels of a target gene, *Dcae*OBP5, in developmental stages (A), tissues (B), and sexes (including adult, antenna, and abdomen of both sexes) (C) were normalized by different reference genes. Bars represent the means and SDs of three biological replicates. Asterisk indicated that *Dcae*OBP5 relative expression normalized by one reference gene or combination of two reference was significant differences among different treatment, and the more asterisks, the greater the difference (*P* < 0.05, Duncan’s test).

To validate the reliability of selected reference genes, the relative expression level of a target gene *Dcae*OBP5 was normalized by the combination of recommended genes by comprehensive evaluation, the top-ranked gene, and the least stable genes as determined by each algorithm. The results were shown as follows: in developmental stages, the expression levels of *Dcae*OBP5 normalized by the least stable reference gene G6PDH were all obviously lower than the results that were normalized by the combination of recommended genes (ACT/RPS3a/RPL13a/β-TUB/ EF1α), by ACT only, and by β-TUB only in all samples except in female pupa. Although the *Dcae*OBP5 expression using RPL13a had no difference with the results normalized using G6PDH in female adult and female pupa, the expression tendency was similar with the combination of ACT/RPS3a/RPL13a/β-TUB/ EF1α, ACT only, and β-TUB only (Fig. 6A). In tissues, the expression levels of *Dcae*OBP5 normalized by stable reference genes of ACT combined with EIF4A, ACT only, and EIF4A only were obviously different with normalization by unstable reference gene of β-TUB in thorax, abdomen, leg, and wing. Moreover, only *Dcae*OBP5 expression normalized by ACT was obviously higher than when the results were normalized by β-TUB in antenna (Fig. 6B). In sexes, the normalized expression results of *Dcae*OBP5 determined using stable reference genes of ACT/EF1α/RPS3a in combination and EF1α only were both different compared with the results normalized by the least stable gene of β-TUB in all the samples. The expression level of *Dcae*OBP5 normalized by ACT was significantly higher than using the least stable reference gene of β-TUB in all samples except male abdomen, but the expression tendency in the abdomen was similar with the ACT/EF1α/RPS3a combination and EF1α only (Fig. 6C).

## Discussion

Currently, qRT-PCR is one of the most convenient, efficient, and widely used tools for measuring the expression levels of target genes. However, the evaluation of reference genes could be an essential step to normalize the quantity of target gene expression and ensure reliability of data (Radonić et al. 2004, Kozera et al. 2013). The ideal reference gene used for normalization in qRT-PCR assays should be steadily transcribed in any conditions, no matter whether among different species, varieties, tissues, abiotic, and biotic stresses, etc. Unfortunately, it is hard to select such a perfect reference gene because it may always vary as the species and experimental conditions changes. In Lepidoptera insects, such as *Plutella xylostella* (L.) (Lepidoptera, Plutellidae) (Fu et al. 2013), *Danaus plexippus* (Lepidoptera, Nymphalidae) (Pan et al. 2015), *Sesamia inferens* (Walker) (Lepidoptera, Noctuidae) (Lu et al. 2015), *Thitarodes armoricanus* (Oberthur) (Lepidoptera, Hepialidae) (Liu et al. 2016), *Helicoverpa armigera* (Hübner) (Lepidoptera, Noctuidae) (Chandra et al. 2017), and *Chilo suppressalis* (Walker) (Lepidoptera, Pyralidae) (Xu et al. 2017), the optimal reference genes in developmental stages and tissues were shown to have significant differences among different species. *Diaphania caesalis*, an important pest in Artocarpus, the identification and validation of reference genes in different treatments have yet been investigated. Therefore, in this study, eight candidate reference genes ACT, β-TUB, GAPDH, G6PDH, RPS3a, RPL13a, EF1α, and EIF4A selected from the *D. caesalis* tanscriptome were assessed under differential stages, tissues, and sexes conditions.

To obtain reliable evaluation and avoid the selection of co-regulated transcripts, a comparison of three mathematical models (geNorm, NormFinder, and Bestkeeper) were used to estimate the stability of these eight genes. The results showed that the best reference genes recommended by different algorithms were not exactly the same under the same treatment condition, which was also found in previous results of other insects and most likely due to different analytical procedures for each program (Qu et al. 2018, Zhang et al. 2018). In our results, geNorm program identified that ACT and RPS3a were the most two stable reference genes according to their M value in developmental stages. However, NormFinder algorithms showed that RPL13a was the best reference gene. For Bestkeeper analysis, the suitable reference genes estimated by SD value was β-TUB, while it was ranked fourth and seventh in geNorm and NormFinder, respectively. Moreover, EIF4A was evaluated as the most stable gene in tissues by Bestkeeper, but it was not the best reference gene recommended by geNorm and NormFinder. Hence, the combined use of these algorithms could ensure more reliable results.

It is worth noting that geNorm provides the suitable number of reference genes based on the pairwise variation (V_n_/V_n+1_) in the case of higher reference gene variability caused by more complex sample sets, it suggests that no extra reference genes are required for normalization when the V_n_/V_n+1_ value below 0.15. However, in our study, almost all the V_n_/V_n+1_ were more than 0.15 except V_5_/V_6_ in developmental stages. It means that multiple reference genes would be needed for normalizing expression values of target gene. However, relevant studies reported that application of multiple reference genes will likely increase the experimental instability and complexity (Kong et al. 2015, Zhang et al. 2018). Fu et al. (2013) demonstrated that the stability of a multi-gene normalizer may decline after adding a relatively unstable reference gene and a combination of the three best reference genes that were recommended as adequate for tissue samples. Based on our results, the lowest V_n_/V_n+1_ value means that the n suitable number of reference genes were needed for normalization, although the the lowest V_n_/V_n+1_ value was above 0.15. One more or one less reference gene would make the quantitative results unstable. Thus, we decided the best number of reference genes according to the lowest V_n_/V_n+1_ value. The choice of reference genes for each treatment complied with the following principle that we began to choose the reference genes from the top-ranked gene of each algorithm until the number of reference genes according with the number of lowest V_n_/V_n+1_ value recommended was reached. Therefore, we concluded that ACT, RPL13a, β-TUB, RPS3a, and EF1α were selected as the suitable reference genes for the developmental stages, ACT and EIF4a were considered as the best internal control for tissues, and three suitable genes of ACT, EF1α, and RPS3a were identified as the best reference genes for sexes.

To validate the selected reference genes, the expression level of a target gene associated to phylogenetic and physiological mechanism should be normalized by the recommend reference gene using qRT-PCR techology. Odorant binding proteins (OBPs) are located in olfactory sensory neurons distributed in antenna, proboscis, and legs in insects, they play crucial role in olfactory mechanism of insects (Archunan 2018). Their function is to bind to the odorant molecule dispersed in the environment and transport them to olfactory receptors, However, in recent years, they have been reported to be endowed with different functions in non-sensory organs of the insect body, such as pheromone delivery, solubilization of nutrients, development, and insecticide resistance (Pelosi et al. 2018). Based on the transcriptome data of *D. caesalis*, 13 transcripts were predicted to code OBPs. We named them as *Dcae*OBP1~OBP13 (unpublished data). Among them, *Dcae*OBP5 was with higher expression level in antenna in the preliminary experiment. So *Dcae*OBP5 were selected to verify the reliability of selected reference genes. The result showed that the recommended reference genes were reliable by validation of the expression level of *Dcae*OBP5.

From the above results, we found two categories of internal reference genes, EF1α and RPS3a, which have been recently widely used as internal control for qRT-PCR in other species or treatments, were also recommended as reference genes for most samples in *D. caesalis*. Recently, ribosomal proteins including RPL5, RPL13A, RPL32, RPL7A, RPS13, and RPS17 have recently been assumed to be stable reference genes for qRT-PCR in many insect species. For example, Shu et al. (2018) proved that RPL13A and RPL7A were demonstrated as the most stable genes in larva and fat body samples of *Spodoptera litura*, respectively. RPL13A was considered to be stable for tissues, developmental stages, and sexes in *Aphidius gifuensis* (Ashmead) (Hymenoptera, Aphidiidae) and for low-temperature treatments in *T. armoricanus* (Liu et al. 2016, Gao et al. 2017). Other ribosomal proteins, such as RPS13, were also verified as stable genes for examination of gene expression profiles in different tissues in *Mythimna separata* (Walker) (Lepidoptera, Noctuidae) (Li et al. 2018), RPL32, and RPS17 for late embryos in *A. albopictus* (Dzaki et al. 2017), and RPL5 and RPS18 for insect life stages in *Bactericera cockerelli* (Sulc) (Homoptera, Psyllidae) (Ibanez et al. 2016). Similarly, our results demonstrated that RPS3a was consistently stably expressed under developmental stages and between sexes in *D. caesalis*. Moreover, the conserved nuclear gene elongation factor 1 alpha (EF1α) has been widely used as the stable reference gene and as a higher-level phylogenetic marker in insects, as recently shown in *Aphis glycines* (Matsumura) (Homoptera, Aphididae), *Chrysomya megacephala* (Fabricius) (Diptera, Calliphoridae), *Harmonia axyridis* (Pallas) (Coleoptera, Coccinellidae) and Psyllid families (Bansal et al. 2012, Wang et al. 2015b, Martoni et al. 2017, Qu et al. 2018). Furthermore, it could also be used as an internal control for the cuticle in *Spodoptera litura* (F.) (Lepidoptera, Noctuidae) (Shu et al. 2018), and for RNAi treatments in *Coccinella septempunctata* (L.) (Coleoptera, coccinellidae) (Yang et al. 2016). In our results, EF1α was also identified as the most stable gene under different developmental stages and between sexes in *D. caesalis*. As two classical reference genes, although Actin and β-TUB were identified as unstable reference gene in some Lepidoptera insects such as grassland caterpillars (Zhang et al. 2017), *C. suppressalis* (Xu et al. 2017) and *Heliconius numata* (Cramer) (Lepidoptera, Nymphalidae) (Prunier et al. 2016), they were still proven to be the stable reference genes in most experimental samples of *D. caesalis*.

A striking finding of this study was that the traditional reference genes, such as GAPDH, were considered as the least stable reference genes in all three treatments, although they have been commonly used as internal controls in much expression research (Purohit et al. 2016, Martoni et al. 2017, Li et al. 2018). Therefore, all these results suggest that each experiment should investigate the normalized reference genes for a specific requirement and condition rather than adopting reference genes from other studies. These results further prove the importance of validating the expression stability of reference genes.

To our knowledge, the present study is the first report on stable evaluation of reference genes in *D. caesalis*. This study demonstrated that the expression stability of reference genes from *D. caesalia* varied across different experimental conditions. Our research represents the critical first step to normalize qRT-PCR data for the accuracy of target genes and further research on phylogenetic and physiological mechanism at the transcriptional level in *D. caesalia*.

## Funding

This work was supported by grants from Hainan provincial Natural Science Foundation (319QN316) and Central Public-interest Scientific Institution Basal Research Fund for Chinese Academy of Tropical Agricultural Sciences (1630142019004).
